# Prognostic and predictive value of circulating tumor cell analysis in colorectal cancer patients

**DOI:** 10.1186/1479-5876-10-222

**Published:** 2012-11-13

**Authors:** Andreia de Albuquerque, Ilja Kubisch, Ulrich Stölzel, Dominikus Ernst, Joachim Boese-Landgraf, Georg Breier, Gudrun Stamminger, Nikos Fersis, Sepp Kaul

**Affiliations:** 1 Department of Molecular Biology, Zentrum für Diagnostik am Klinikum Chemnitz, Flemmingstrasse 2, 09116, Chemnitz, Germany; 2 Department of Internal Medicine, Klinikum Chemnitz, Chemnitz, Germany; 3 Department of Surgery, Klinikum Chemnitz, Chemnitz, Germany; 4 Department of Pathology, Technischen Universität Dresden, Dresden, Germany; 5 Zentrum für Diagnostik, Chemnitz, Germany; 6 Department of Gynecology, Klinikum Chemnitz, Chemnitz, Germany

**Keywords:** Circulating tumor cells, Colorectal cancer, Reverse transcription real-time polymerase chain reaction

## Abstract

**Objective:**

The aim of this study was to assess the prognostic and predictive values of circulating tumor cell (CTC) analysis in colorectal cancer patients.

**Patients and methods:**

Presence of CTCs was evaluated in 60 colorectal cancer patients before systemic therapy - from which 33 patients were also evaluable for CTC analysis during the first 3 months of treatment - through immunomagnetic enrichment, using the antibodies BM7 and VU1D9 (targeting mucin 1 and EpCAM, respectively), followed by real-time RT-PCR analysis of the tumor-associated genes *KRT19*, *MUC1*, *EPCAM*, CEACAM5 and *BIRC5*.

**Results:**

Patients were stratified into groups according to CTC detection (CTC negative, when all marker genes were negative; and CTC positive when at least one of the marker genes was positive). Patients with CTC positivity at baseline had a significant shorter median progression-free survival (median PFS 181.0 days; 95% CI 146.9-215.1) compared with patients with no CTCs (median PFS 329.0 days; 95% CI 299.6-358.4; Log-rank *P* < .0001). Moreover, a statistically significant correlation was also founded between CTC detection during treatment and radiographic findings at the 6 month staging. This correlation applied to CTC results before therapy (odds ratio (OR), 6.22), 1 to 4 weeks after beginning of treatment (OR, 5.50), 5 to 8 weeks after beginning of treatment (OR, 7.94) 9 to 12 weeks after beginning of treatment (OR, 14.00) and overall CTC fluctuation during the course of treatment (OR, 20.57).

**Conclusion:**

The present study provides evidence of a strong correlation between CTC detection and radiographic disease progression in patients receiving chemotherapy for colorectal cancer. Our results suggest that in addition to the current prognostic factors, CTC analysis represent a potential complementary tool for prediction of colorectal cancer patients’ outcome. Moreover, the present test allows for molecular characterization of CTCs, which may be of relevance to the creation of personalized therapies.

## Introduction

Nowadays, systemic treatment of metastatic cancer can result in a modest survival improvement for patients. However, palliation still remains the main treatment goal for cancer patients with metastatic lesions [1]. Many factors have been proposed as useful independent prognostic factors of recurrence and overall survival (OS) in cancer [2-4]. Yet, tumor stage still continues to play a fundamental role in the management of patients as the most powerful and reliable predictor of prognosis [5]. Moreover, it represents the operational basis for choosing the most appropriate therapy and for evaluating the efficacy of different therapeutic methods through the comparison of expected survival rates [6]. Nevertheless, current prognostic factors do often not allow for a more personalized approach in cancer treatment and there is a lack of accurate methodologies to identify patients that are destined to progress quickly or that would benefit from a more aggressive therapy.

We recently demonstrated that circulating tumor cell (CTC) analysis through an in-house optimized assay, using immunomagnetic enrichment, followed by real-time RT-PCR detection of a multimarker gene panel, can be used to isolate and characterize CTCs in a variety of adenocarcinomas [7,8] and be an indicator of shorter disease-free survival in pancreatic cancer patients [9]. From these feasibility studies, we designed a study to evaluate the prognostic and predictive values of CTC analysis in colorectal cancer patients.

## Patients and methods

### Patients selection and PB sampling

Written informed consent was obtained from all participants and the study was approved by the local medical ethical committee. Principal inclusion criteria were as follows: patients with histologically and radiographically proven colorectal cancer, initiating any first- or second-line systemic therapy. Patients with a history of previous malignancy and patients with active infection were excluded.

From all subjects, 10.0 ml whole blood samples were collected before therapy in EDTA tubes (Sarstedt AG & Co, Nümbrecht, Germany) and CTCs were isolated within 4 h of specimen collection.

### Patient follow-up

Patients underwent chemotherapy as appropriate for their diagnosis and had disease evaluation from their medical oncologist according to the institutional guidelines. The evaluation included a physical examination, a complete blood count, blood chemical tests, screening for serum tumor markers, radiography and computed tomographic scan, magnetic resonance imaging, according to tumor type and stage. The planned re-evaluation for patients with metastatic disease was performed every 3 months. Response was evaluated according to clinical criteria codified by the Response Evaluation Criteria in Solid Tumors (RECIST) [10] by a team of medical oncologist and radiologists. Each disease assessment was classified as complete response (CR) partial response (PR), stable disease (SD) or progressive disease (PD). The primary end point for metastatic patients was time to progression. For response to therapy in the metastatic setting, the favourable group was defined as having non progressive (NP) disease (CR, PR and SD categories) and the unfavourable group was defined as that with PD or death.

### CTCs isolation, mRNA extraction, cDNA synthesis and multimarker real-time PCR analysis

CTCs were isolated from PB through immunomagnetic enrichment, using the antibodies BM7 and VU1D9 (targeting mucin 1 and EpCAM, respectively), followed by mRNA extraction, cDNA synthesis and real-time RT-PCR analysis of the tumor-associated genes *KRT19*, *MUC1*, *EPCAM*, CEACAM5 and *BIRC5*. All the methodologies and primers, as well as real-time RT-PCR validation, have already been described elswhere [7-9]. According to the analytic detection limit of our assay, the Cq cut-off, under which a marker gene is considered to be positive, was defined as: 36.0 for *KRT19*, 37.1 for *MUC1*, 36.0 for *EPCAM*, 37.8 for *CEACAM5*, 35.0 for *BIRC5*, 37.3 for *SCGB2A2* and 37.9 for *ERBB2*. A sample was considered to be CTC positive when at least one of the marker genes was positive.

### Statistical analysis

The potential correlation between CTC findings and the clinicopathological characteristics of the patients was tested using either a chi-squared test or a Fisher's exact test.

Progression-free survival (PFS) was defined as the time between the baseline CTC assessment (the initiation of treatment) and the documentation of first radiographic disease progression or death. Patients who were alive and progression free at the time of analysis were censored using the time between the baseline CTC assessment and their most recent follow-up evaluations. PFS in CTC-positive and CTC-negative groups was compared with the Kaplan-Meier method, and differences were tested with the log-rank test.

The association between CTC status during the course of treatment and radiographic images was assessed by logistic regression. Patients were classified into two groups, according to their status after 6 months of initiating a systemic therapy: the favourable group was defined as having non progressive disease (CR, PR and SD categories) and the unfavourable group was defined as that with PD or death. Clinical outcome was then associated with CTC results obtained before therapy, after 1–4 weeks, 5–8 weeks and 9–12 weeks of therapy and with a change in CTC positivity during the course of treatment. Univariate analyses were performed and odds ratios (OR) together with the respective 95% confidence intervals (95% CI) were estimated.

A *P* value < 0,05 was considered to be statistically significant. All analyses were carried out using SPSS (Version 17.0, SPSS, Chicago, IL, USA).

## Results

### Patient characteristics

Between April 2009 and June 2011 a total of 60 colorectal cancer patients, meeting the inclusion and exclusion criteria, were enrolled. During the median follow-up period of 242 days (range 191.8 - 292.2 days), evidence of disease progression was documented in 51 patients and death had occurred in 17 patients. Detailed clinicopathological characteristics of the patients are given in Table 1.

**Table 1:** **Colorectal cancer patients**’ **clinicopathological characteristics**

	**Colorectal Patients**	
**Variable**	**No**.	%
Total No. of patients	60	
Age at study entry, years		
Median	65.2	
Range	40–80	
Gender		
Female	17	28.3
Male	43	71.7
Stage		
II	4	6.7
III	8	13.3
IV	48	80.0
Tumor size		
T1	5	8.3
T2	7	11.7
T3	9	15.0
T4	39	65.0
Lymph nodes		
N0	13	21.7
≥ N1	47	78.3
Histology grade		
G2	39	65.0
G3	16	26.7
G4	5	8.3
Metastasis		
Yes	54	90.0
No	6	10.0

### CTCs at baseline

CTC analysis before treatment revealed that 65.0% of colorectal cancer patients were CTC positive. Positivity rates for each individual marker were as follows: 36.7% for *KRT19*, 23.3% for *MUC1*, 30.0% for *EPCAM*, 26.7% for *CEACAM5* and 16.7% for *BIRC5*.

### Correlation between CTCs and clinicopathological features

The presence of CTCs in the PB of pancreatic patients at baseline did not correlate with gender (p = 0.218), stage (p = 0.152), tumor size (p = 0.216), lymph nodes (p = 0.109) and tumor grading (p = 0.319). However, associations were found between the presence of metastasis and CTC positivity (p = 0.001).

### Correlation between CTCs and PFS in pancreatic cancer patients

**Figure 1:**
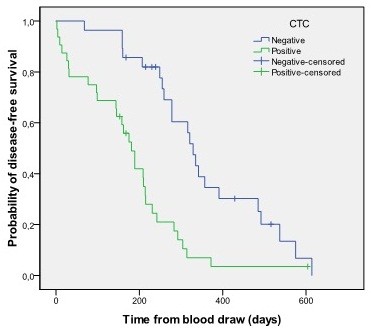
PFS of colorectal cancer patients with and without CTCs in 10 ml of blood before therapy (CTC positivity is defined by when at least one marker gene is positive).

The correlation between PFS and baseline CTCs status in colorectal patients was compared with the Kaplan-Meier method and differences were tested using the log-rank test (Figure 1). PFS was calculated for groups defined by the presence or absence of CTCs before initiating chemotherapy. The overall median PFS for the assessable patients was 242.0 days (95% CI 191.8-292.2). Patients with CTC positivity at baseline had a significant shorter median PFS (181.0 days; 95% CI 146.9-215.1) compared with patients with no CTCs (median PFS 329.0 days; 95% CI 299.6-358.4; Log-rank *P* < .0001).

### Correlation between CTCs and radiographic images

A total of 33 colorectal cancer patients were also evaluable for the analysis of CTC status before therapy and after 1 to 4, 5 to 8 and 9 to 12 weeks of treatment. In this set of patients, 48.5% were classified as having disease progression at the 6 month staging. From the 16 patients with PD or death, 14 (87.5%) were CTC positive before starting a new line of therapy, 12 (75.0%) had positive CTC in their PB at 1 to 4 and 5 to 8 weeks of treatment, and 13 (81.3%) patients were CTC positive at 9 to 12 weeks after starting treatment (Table 2).

**Table 2:** **Type and line of therapy**, **treatment response and CTCs results before and during the course of therapy from 33 colorectal cancer patients**

**PatientID**	**Therapy**	**Line of therapy**	**Staging outcome**	**CTCs before therapy**	**CTCs during therapy**		
					**1**–**4 weeks**	**5**–**8 weeks**	**9**–**12 weeks**
5	FOLFIRI+Beva	2	PD	+	-	-	+
7	FUFOX	2	PD	+	+	+	+
8	FOLFIRI+Beva	2	NP	+	+	+	-
9	FUFIRI	2	PD	+	+	+	+
15	FOLFIRI	1	PD	-	+	+	+
36	FOLFOX-4	1	PD	+	-	+	+
39	FUFIRI	2	PD	+	-	-	-
47	FOLFIRI+Beva	1	NP	+	-	-	-
49	FOLFOX-4	1	NP	+	-	+	-
49	FOLFIRI	2	PD	+	+	+	+
65	Panitumumab	2	NP	-	-	+	-
65	Panitumumab	2	PD	+	+	+	+
70	FOLFOX-4	1	NP	-	-	+	-
73	FOLFOX-4	1	PD	+	-	-	+
74	FOLFIRI+Beva	2	PD	+	+	+	+
77	AIO+Beva	1	NP	+	+	+	-
80	FOLFIRI+Beva	1	NP	-	-	-	-
81	FUFIRI	1	PD	-	+	+	+
81	FUFOX	2	PD	+	+	+	+
87	AIO	1	NP	+	-	-	+
91	AIO +Beva	2	NP	-	-	-	-
97	FOLFIRI +Beva	1	NP	+	+	+	+
97	FOLFIRI +Beva	1	PD	+	+	+	-
99	FOLFOX-4	1	NP	-	-	-	-
109	AIO	1	PD	+	+	+	+
112	FOLFIR+Beva	1	NP	-	+	-	-
139	FOLFIR+Beva	1	NP	+	+	-	-
150	AIO +Beva	1	NP	-	-	-	-
151	FUFOX	1	NP	+	+	-	+
151	FUFOX	1	PD	+	+	+	+
171	FOLFOX-4	1	NP	+	-	-	-
184	AIO	1	NP	-	-	-	-
195	AIO	1	PD	+	+	+	+

The predictive value of CTC analysis before therapy and after 1 to 4, 5 to 8 and 9 to 12 weeks of treatment, as well as the overall fluctuation of CTC positivity during the course of treatment is shown in Table 3. Significant associations were found between CTC analysis at all time points and radiographic outcome at 6 month restaging, being CTC fluctuations the variable with the strongest correlation. That is, patients with an increase in the number of positive marker genes, during the course of therapy, had 20.57 times the odds of radiographic disease progression compared with patients who had no CTCs or a decrease in CTC positive markers.

**Table 3:** The predictive value of CTCs before and during the course of treatment in colorectal cancer patients when compared with 6 months clinical outcome assessed by radiographic images

**Variable**	**Odds ratio** (**95%CI**)	***P***
CTCs before therapy (pos vs neg)	6.22 (1.07-36.21)	0.042
CTCs after 1–4 weeks of therapy (pos vs neg)	5.50 (1.22-24.81)	0.027
CTCs after 5–8 weeks of therapy (pos vs neg)	7.94 (1.60-39.42)	0.011
CTCs after 9–12 weeks of therapy (pos vs neg)	14.00 (2.60-75.41)	0.002
CTCs fluctuations during therapy (increase *vs* decrease)	20.57 (2.17-194.95)	0.008

## Discussion

To the best of our knowledge, here we report the first study using mucin 1- and EpCAM-based immunomagnetic enrichment, followed by real-time RT-PCR analysis of *KRT19*, *MUC1*, *EPCAM*, CEACAM5 and *BIRC5*, as a way to detect and evaluate the prognostic and predictive effect of CTCs in PB of colorectal cancer patients.

As treatment has become more effective for colorectal cancer, decision making has also become more complicated. Five classes of drugs are currently available for treatment and therefore selection and monitoring of therapy have become more difficult [11]. Standard practise is to change treatment after several weeks or months of therapy if there is evidence of progression. However, after initiation of systemic treatment, current methodologies do not often allow for an accurate and early assessment of clinical benefit. Thus, patients may be either treated for prolonged periods with an inactive therapy or a potentially active therapy may be discontinued prematurely [1]. Therefore, we have evaluated whether CTC status, before and during treatment, are indicative of treatment benefit in advance of radiographic changes.

In the present study, we report the results of an in-house immunomagnetic/real-time RT-PCR assay for the detection and characterization of CTCs in colorectal cancer patients. Assay development and validation (addressing typical technical concerns, such as specificity, contaminants, efficiency, sensitivity and sample quality), are extensively explained elsewhere [7-9]. By applying this methodology, our results show a correlation between CTC assessments and radiographic determinations of disease progression in patients receiving chemotherapy for metastatic colorectal cancer. This correlation applies not only for CTC results obtained during the treatment, but also to CTC assessments obtained as far in advance as 6 months before imaging. Moreover, fluctuations in CTC levels during the course of treatment were also associated with tumor responsiveness determined by radiographic imaging. Patients with an increase in the number of positive markers, during the course of therapy, had 20.57 times the odds of radiographic disease progression compared with patients who had no CTCs or a decrease in CTC positive markers. Therefore, our findings suggest that serial CTC assessments taken before and during the first 3 months of treatment, used in conjunction with imaging, could help to confirm evidence of tumor response or decide doubtful findings.

Analysis of CTCs in blood of cancer patients can be performed by several rare cell detection techniques and promising results with potential clinical relevance have been obtained. One of the largest published studies on CTC detection in metastatic colorectal cancer, involving 430 patients, was performed by Cohen et al. [12]. Patients had their blood collected before treatment and at four different points during treatment schedule and CTCs were detected using the CellSearch® System. Patients were stratified according to favourable (< 3 CTCs per 7.5 ml of blood) or unfavourable (≥ 3 CTCs per 7.5 ml of blood) CTCs counts. The study showed that, at all time points, median PFS and OS rates were twice as high for patients in the favourable group as compared to those in the unfavourable group. The study also found a significant prognostic correlation between patient grouping by image response and CTCs, meaning that if patients presented elevated CTCs after therapy, they were more likely to have a worse prognosis. An update of this study, 1 year later and enclosing extended follow-up times, reported a pronounced PFS and OS difference between favourable and unfavourable groups, mostly in patients receiving first-line therapy [13].

Our study, even if using another CTC detection method and a smaller cohort of patients, confirms not only the previous findings by Cohen et al., but gives also extra information about CTC phenotypes. To date, a variety of research methods have been developed to isolate and enumerate CTCs. The existing CTC detection assays rely on various properties of CTCs, with each one having unique advantages and limitations. However, no enrichment or detection method has yet proven to be the golden standard and continuing efforts are needed to improve the reliability of CTC detection techniques. Even if the CellSearch® System has been validated via multicenter studies and is the only FDA cleared device for enumeration of CTCs, its use presents some limitations that may be crucial for elucidating and optimizing the use of CTCs in cancer management. By simply enumerating tumor cells, the CellSearch® System is missing information regarding the enormous biological and clinical contributions that CTCs can provide [14]. Therefore the future of rare tumor cell analysis relies also in the development of sensitive and relatively inexpensive assays capable of generating CTC molecular profiles, which may allow for the identification of subgroups of patients who would benefit from a specific therapy.

Nevertheless, limitations of this work must be considered. The study population was relatively small which may influence the interpretation of the results. However, small well-designed studies are of great value once that they can provide results quickly and becoming part of a preliminary selection in order to further design larger confirmatory studies. Patients also had flexibility regarding the exact dates of blood draws and computed tomography scans. However, the time frames for data analysis were well defined and this flexibility reflects the everyday in clinical practice.

## Conclusion

Our results suggest that the optimal cancer staging system should include both anatomic and non anatomic factors, such as CTCs. In addition to strictly tumor-related descriptors, there are also genetic characteristics (gene expression profiling patterns, clustering of genetic alterations and multimarker phenotypes) that represent potential complementary tools for tumor classification. These tests could become an integral part of a future staging system, in association with more traditional morphologic features, able of enhancing the prediction of patient outcome and ultimately allowing tailored therapy and improved patient care. The present study provides not only evidence of a strong correlation between CTC detection and radiographic disease progression in patients receiving chemotherapy for colorectal cancer, but offer also the possibility of molecular characterization of CTCs, which may imperative to the creation of personalized therapies.

## Competing interests

The authors declare that they have no competing interests.

## Authors’ contributions

AdA performed the experimental work and statistical analysis, contributed for study design, data interpretation and drafted the manuscript. SK contributed for the experimental work, study design and data interpretation. GB contributed for critical revision of statistical analysis and of the manuscript. IK, DE, US and JB selected the patients for blood drawing and provided the patient data. GS and NF critically revised the manuscript. All authors read and approved the final manuscript.
